# Immunohistochemical Evaluation of the Tumor Immune Microenvironment in Pancreatic Ductal Adenocarcinoma

**DOI:** 10.3390/diagnostics15050646

**Published:** 2025-03-06

**Authors:** Gelu Mihai Breaza, Raluca Maria Closca, Alexandru Cristian Cindrea, Florin Emil Hut, Octavian Cretu, Laurentiu Vasile Sima, Marina Rakitovan, Flavia Zara

**Affiliations:** 1 Department of Microscopic Morphology, University of Medicine and Pharmacy “Victor Babes”, 300041 Timisoara, Romania; gelu.breaza@umft.ro (G.M.B.); marina.rakitovan@umft.ro (M.R.); flavia.zara@umft.ro (F.Z.); 2 University Clinic of Surgery I, University of Medicine and Pharmacy “Victor Babes”, 300041 Timisoara, Romania; florin.hut@umft.ro (F.E.H.); octavian.cretu@umft.ro (O.C.); sima.laurentiu@umft.ro (L.V.S.); 3 Department of Pathology, Emergency City Hospital, 300254 Timisoara, Romania; 4 Department of Surgery, University of Medicine and Pharmacy “Victor Babes”, 300041 Timisoara, Romania; alexandru.cindrea@umft.ro; 5 Emergency Department, Emergency Clinical Municipal Hospital, 300079 Timisoara, Romania; 6 Center for Hepato-Bilio-Pancreatic Surgery, University of Medicine and Pharmacy “Victor Babes”, 300041 Timisoara, Romania; 7 Oro-Maxillo-Facial Surgery Clinic, Emergency City Hospital, 300062 Timisoara, Romania

**Keywords:** pancreatic ductal adenocarcinomas, tumor immune microenvironment, immunohistochemistry

## Abstract

**Background**: Pancreatic ductal adenocarcinoma is an aggressive neoplasm with a complex carcinogenesis process that must be understood through the interactions between tumor cells and tumor microenvironment cells. **Methods**: This study was retrospective with a chronological extension period of 16 years and included 56 cases of pancreatic ductal adenocarcinoma. This study identified, quantified, and correlated the cells of the tumor immune microenvironment in pancreatic ductal adenocarcinoma with major prognostic factors as well as overall survival, using an extensive panel of immunohistochemical markers. **Results**: Three tumor immunotypes were identified: subtype A (hot immunotype), subtype B (intermediate immunotype), and subtype C (cold immunotype). Patients with immunotype C exhibit considerably higher rates of both pancreatic fistulas and acute pancreatitis. Immunotypes B and C significantly increased the risk of this complication by factors of 3.68 (*p* = 0.002) and 3.94 (*p* = 0.001), respectively. The estimated probabilities of fistula formation for each immunotype are as follows: 2.5% for immunotype A, 25% for immunotype B, and 28% for immunotype C. There was a statistically significant difference in median survival times according to tumor immunotype (*p* < 0.001). Specifically, patients with immunotype C tumors had a median survival time of only 120.5 days, compared to 553.5 days for those with immunotype A and 331.5 for immunotype B tumors. **Conclusions**: The identification of the immunotype of pancreatic ductal adenocarcinoma can be a predictive factor for the occurrence of complications such as pancreatic fistula as well as for overall survival.

## 1. Introduction

Pancreatic adenocarcinoma is an extremely aggressive form of neoplasia that arises from the exocrine component of the pancreas and accounts approximately 90% of pancreatic cancers [1]. Its incidence has doubled in the last two decades and is four times higher in countries with a high Human Development Index [2,3]. Patients present with non-specific and insidious symptoms such as epigastric pain radiating to the back, weight loss, nausea, fatigue and, if the location of the tumor is in the pancreatic head, jaundice [4].

Alcohol consumption, cigarette smoking, and type 2 diabetes have been identified as high-risk factors in the pathogenesis of pancreatic adenocarcinoma [5,6]. Many studies indicate that diabetes doubles the risk of pancreatic carcinoma and cigarette smoking for 50 pack-years increases the risk by 91% [5,7]. Also, a family history of pancreatic cancer, obesity, and chronic pancreatitis are risk factors linked to pancreatic cancer [8]. Certain genetic mutations that could also contribute to the diagnosis of pancreatic cancer include those associated with *BRCA1*, *BRCA2*, *PALB2*, and *ATM* genes [9]. Studies show that the sequence in sporadic pancreatic carcinoma is characterized by hyperplastic changes in the ductal epithelium, atypical ductal hyperplasia, and invasive ductal adenocarcinoma. This period is orchestrated by a sequence of genetic changes that include the KRAS and Her-2 genes, followed by p-16 alteration and finally gene mutations in p53, DPC4 and BRCA2, and other tumor suppressor genes. The presence of KRAS mutation in exocrine pancreatic secretions has been frequently observed in patients with pancreatic cancer and also in patients with chronic pancreatitis before the development of pancreatic adenocarcinoma [10].

Kolbeinsson et al. showed that 20% of patients have a resectable tumor at diagnosis and a median survival rate of 12.6 months. In contrast, the median survival rate was 3.5 months for patients for whom surgery was not a therapeutic option. A total of 55% of patients had already progressed to metastatic disease [11]. The 5-year overall survival rate for pancreatic adenocarcinoma remains under 10.8% for both metastatic and resectable tumors [12]. Mortality is on the rise, increasing by about 1% annually and is expected to become the second leading cause of cancer death by 2030 [13,14,15].

The standard approach for patients with a resectable tumor is surgery followed by chemotherapy. Radiotherapy is considered the standard procedure in distant metastases that cannot be completely resected. Patients with locally advanced tumors are given systemic therapy followed by radiation and patients with advanced tumors are given a combination of non-surgical interventions, including multiagent chemotherapy regimens [16,17,18].

The pathogenesis and progression of pancreatic adenocarcinoma is orchestrated by a complex tumor immune microenvironment which facilitates metabolic changes and interacts with tumor cells [1]. The current methodology to assess the tumor microenvironment is deficient and involves manual morphological identification and reporting of perineural and lymphovascular invasion on histological sections [19]. The diverse cell types of the tumor immune microenvironment are not identified, quantified, and reported in standard pathological reports. However, the use of targeted therapies requires a personalized assessment of the characteristics of the immune microenvironment of each tumor.

The aim of this study was to identify and quantify the cells of the tumor immune microenvironment of pancreatic ductal adenocarcinoma using an extensive panel of immunohistochemical markers and to correlate them with overall survival and with prognostic factors.

## 2. Materials and Methods

### 2.1. Patient Selection and Inclusion Criteria

**Figure 1:**
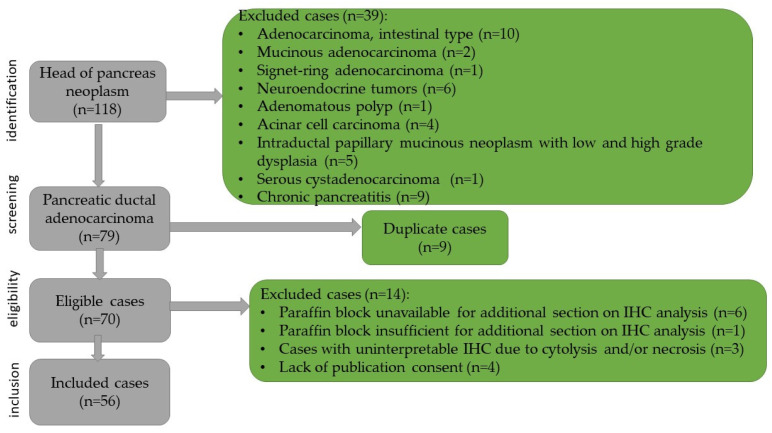
Flowchart of the case selection.

This study was retrospective, with chronological extension over a period of 16 years, and included the cases of 56 pancreatic ductal adenocarcinoma. The patients were admitted to the Department of General Surgery of the Timisoara’s Emergency City Hospital between January 2008 and December 2023. The case identification was performed using the specimen reception registers of the Department of Pathology. The cases were integrated into the clinical context using the computer database of the hospital. For each patient, age, sex, symptoms, size of the lesion, imaging aspects, surgical procedure, postoperative complications, tumor stage, recurrence, and oncological treatment were identified. The data related to the survival of the patients were obtained from the Central Register of the Population Records. The inclusion criteria for the study were as follows: patient’s age over 18 years, resectable pancreatic tumor, primary pancreatic tumor, pancreaticoduodenectomy specimen, ductal pancreatic adenocarcinoma confirmed by histopathologic examination, negative resection margins, and paraffin block available and sufficient for immunohistochemical staining. The case selection method as well as the exclusion criteria are shown in Figure 1.

Data regarding patients’ demographics (sex, age, date of admission, and date of death), surgical procedures (usage of preoperatory biliary stents and surgical technique—modified or classic), complications (pancreatic, biliary or enteral fistulas, and acute pancreatitis), histological grading, and tumor staging have been collected using Microsoft Excel (Microsoft Office 16, Albuquerque, NM, USA).

### 2.2. Ethical Considerations

This study was performed relying on the Romanian legislation and the ethical guidelines of the Declaration of Helsinki. The informed consent of the patient was performed, and the presence of the annex that accompanied the biopsy was secured. The histological slides were processed in accordance with the Ministry of Health recommendations and international protocols. The Institutional Review Board Statement No. E-494/07.02.2025 was obtained by the ethical commission of Timisoara’s Emergency City Hospital.

### 2.3. Laboratory Technique

The harvested specimens were fixed in 4% *v*/*v* buffered formaldehyde. Four-micrometer-thick sections were cut using a semi-automated Leica RM2235 rotary microtome (Leica Biosystems, Nussloch, Germany) and displayed on SuperFrost™ microscope slides (St. Louis, MO, USA). For morphological diagnosis, the hematoxylin–eosin technique was primarily used. The microscopical aspects were recorded for each tumor as follows: architectural pattern of growth, cells type, rate of mitotic figures counted per 10 high-power fields, necrosis, inflammatory response, angiolymphatic invasion, perineural invasion, and assessment of the resection margins.

For the evaluation and quantification of the tumor immune microenvironment, the immunohistochemical reactions were used. All the steps of the immunohistochemical reactions were performed with Leica Bond-Max automatic device (Leica Biosystems Melbourne Pty Ltd., Waverley, Australia), using the standardized and recommended protocol of the manufacturer. All the antibodies and reagents for immunohistochemical staining were purchased from Leica Biosystems, Newcastle, UK. The characteristic data of the immunohistochemical reagents, regarding the type of antibodies, clone, and dilution, are presented in Table 1. Sections for immunohistochemistry were performed sequentially, and each tumor slice was stained with a single primary antibody and had a thickness of two micrometers. The evaluation was performed manually and independently by two experienced pathologists, who evaluated 10 microscopic fields at 40× for each section. Interindividual variations were corrected by subsequent discussion and agreement.

Immune cells were calculated as a percentage of the total number of cells (tumor, stromal, and immune). Based on this percentage, the immune cellular infiltrate was stratified into three categories, using the following cutoffs: marked (over 50%), moderate (10–50%), and discreet (below 10%). Also, LCA-positive lymphocytes were calculated as a percentage of the total number of immune cells, and B and T lymphocytes (CD20, CD3, CD4, CD5, and CD8 positive) as a percentage of the total number of LCA-positive lymphocytes. CD117 is expressed not only on mast cells, but also on dendritic cells progenitors. Additionally, morphological criteria was used to identify mast cells (round cells with a lobulated nucleus and dense basophilic granule in the cytoplasm), as well as the CD1a marker that highlighted progenitor dendritic cells.

For the microscopic evaluation of stromal fibrosis, Masson’s trichrome staining was performed using the complete staining kit from Bio-Optica, Milano spa, REF 04-010802, lot 24190 (20134, Milano, Italy). Collagen fibers were identified in the areas of fibrosis with blue color. Fibrosis was quantified as a percentage of the total area examined per field at 40× objective, and tumors were reported with mild (less than 10%), moderate (10–50%), and severe (more than 50%) fibrosis.

### 2.4. Endpoints

The primary endpoint was the identification and quantification of the tumor immune microenvironment, and the second was to evaluate the association between the main descriptive findings and the major prognostic factors as well as overall survival.

### 2.5. Statistical Plan

Descriptive statistics are presented as the median and interquartile range (IQR) for continuous variables, and as absolute numbers and percentages for categorical variables. Data normality was assessed using the Kolmogorov–Smirnov test. Where necessary, further analysis has been conducted using graphical representations (histograms) and goodness-of-fit criteria (Akaike Information Criterion (AIC)). Practically, the maximum likelihood estimation was calculated for each type of distribution. Since all variables deviated from normality, group comparisons were conducted using the non-parametric Kruskal–Wallis test. Differences in the prevalence of categorical variables between groups were evaluated using the Chi-square test.

Logistic regression models were generated to assess the relationships between risk factors and the rate of developing certain complications. The AIC was used as the selection criteria for the best-fit model. Further analysis of the complications includes Monte Carlo simulations with 10,000 samples. The simulated data frame comprised patients’ age (using Weibull distribution), sex, grade of the disease, stage, and type. The probability of developing pancreatic fistulas has been calculated based on the initial logistic regression model.

Kaplan–Meier survival curves (with log-rang testing) were generated to analyze survival probabilities for each tumor type, while overall survival times were computed for the survival analysis at 28 days, 1, 2 and 3 years.

Data analysis has been conducted utilizing R software v4.3.2. Packages “dplyr”, “ggplot2”, “fitdistrplus”, “MASS”, “rcompanion”, “rms”, “survival”, “survminer”, and “viridis” have been used.

## 3. Results

### 3.1. Histopathological Findings

The microscopic examination in hematoxylin–eosin staining identified 56 cases of pancreatic ductal adenocarcinoma, most of which were moderately differentiated G2 (87.5%). The immunohistochemical study of the tumor immune microenvironment revealed three tumor subtypes: subtype A (hot immunotype), subtype B (intermediate immunotype), and subtype C (cold immunotype). Table 2 presents the detailed immunohistochemical profile of the tumor immune microenvironment.

**Figure 2:**
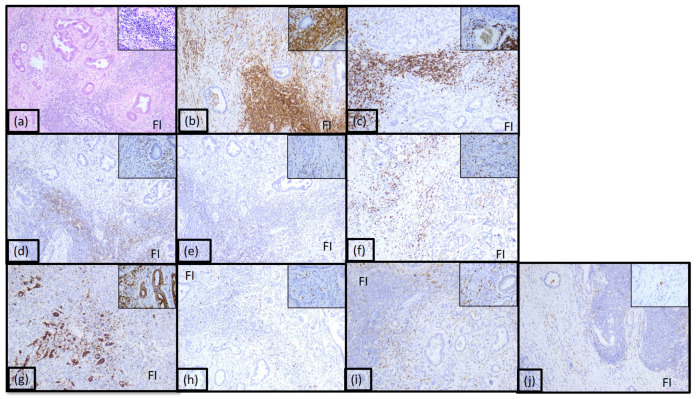
Morphological aspect and immunohistochemical profile of the pancreatic ductal adenocarcinoma immunotype A, ob.10× (ob.40× in the corner): (**a**) hematoxylin-eosin staining; (**b**) LCA; (**c**) CD20; (**d**) CD3; (**e**) CD4; (**f**) CD8; (**g**) CD5; (**h**) CD117; (**i**) CD68; (**j**) CD1a; FI: front of invasion.

The majority of the tumors (39%) were classified as immunotype A (Figure 2 and Supplemental Figure S1). This immunotype was characterized by a rich inflammatory infiltrate arranged both peri- and intratumorally and called it the hot immunotype. The tumoral immune microenvironment was predominantly represented by lymphocytes (60% of the total inflammatory infiltrate), mostly arranged in the front of invasion. CD20 positive B lymphocytes were numerous, representing 30–35% of all lymphocytes. CD4 T lymphocytes represented 10%, and CD8 positive T lymphocytes represented 5%. The CD4:CD8 ratio was 2 for the majority of tumors in this category. Numerous positive CD3 and CD5 T lymphocytes were also identified (30% and 20%, respectively). The rest of the tumor immune microenvironment was represented by CD68 positive macrophages (20%) and mast cells (10%). A few dendritic cells presenting antigen CD117 positive (5%) were also identified, as well as neutrophil granulocytes (5%), located predominantly in the front of invasion.

**Figure 3:**
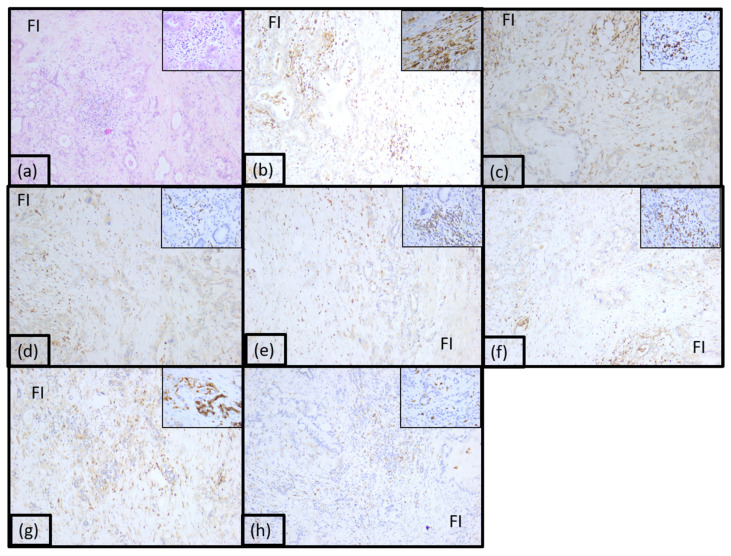
Morphological aspect and immunohistochemical profile of the pancreatic ductal adenocarcinoma immunotype B, ob.10× (ob.40× in the corner): (**a**) hematoxylin-eosin staining; (**b**) LCA; (**c**) CD20; (**d**) CD3; (**e**) CD4; (**f**) CD8; (**g**) CD5; (**h**) CD117.

Twenty-five percent of adenocarcinomas were classified as immunotype B (Figure 3 and Supplemental Figure S1). This immunotype was characterized by a moderate inflammatory infiltrate mostly arranged diffusely in the front of invasion and focally intratumorally and was called the intermediary immunotype. The tumoral immune microenvironment was represented by lymphocytes (55% of the total inflammatory infiltrate). CD20 positive B lymphocytes were low, representing 15% of all lymphocytes. CD4 T lymphocytes represented 10%, and CD8 positive T lymphocytes represented 40%. The CD4:CD8 ratio was 1:4 for the majority of tumors in this category. CD3 and CD5 positive T lymphocytes were also identified (15% and 20%, respectively). The rest of the tumor immune microenvironment was represented by CD68 positive macrophages (20%) and plasma cells (10%). A few dendritic cells presenting antigen CD117 positive (5%) were also identified, as well as neutrophil granulocytes (5%) and eosinophils (5%), located predominantly in the front of invasion and perivascular.

**Figure 4:**
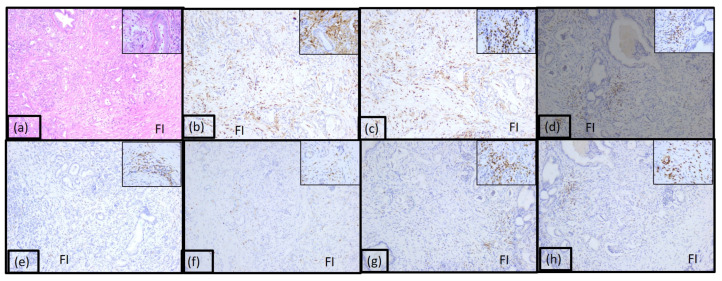
Morphological aspect and immunohistochemical profile of the pancreatic ductal adenocarcinoma immunotype C, ob.10× (ob.40× in the corner): (**a**) hematoxylin-eosin staining; (**b**) LCA; (**c**) CD20; (**d**) CD3; (**e**) CD4; (**f**) CD8; (**g**) CD5; (**h**) CD117.

The immunotype C named cold immunotype constituted 36% of case and was characterized by poor inflammatory infiltrate both peri and intratumorally (Figure 4 and Supplemental Figure S1). The tumoral immune microenvironment was represented by numerous lymphocytes (90% of the total inflammatory infiltrate). CD20 positive B lymphocytes were numerous, representing 60% of all lymphocytes. CD4 T lymphocytes represented 5%, and CD8 positive T lymphocytes represented 20%. The CD4:CD8 ratio was 1:4 for the majority of tumors in this category. CD3 and CD5 positive T lymphocytes were also identified (5% and 10%, respectively). The rest of the tumor immune microenvironment was represented by plasma cells (10%) and few dendritic cells presenting antigen CD117 positive.

**Figure 5:**
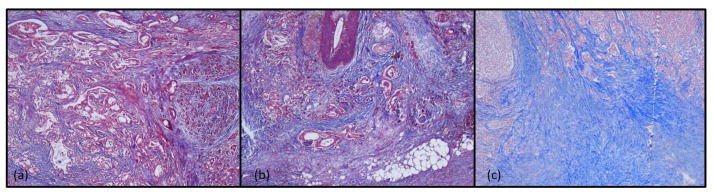
Histochemical aspects of tumor immunotypes in Masson’s trichrome staining, ob.5×: (**a**) immunotype A; (**b**) immunotype B; (**c**) immunotype C.

Masson’s trichrome staining revealed mild fibrosis in immunotype A tumors, moderate fibrosis in immunotype B tumors, and marked fibrosis in immunotype C tumors, both in the intra and peritumorally areas (Figure 5).

### 3.2. Descriptive Statistics

The final dataset comprises 56 patients with complete data. The patients were divided into three groups based on the histopathological reasons described above. The majority were male (57.1%), and the overall median age was 64 years, as shown in Table 3. More than half of the patients received a stent (51.8%), and the modified surgical technique was the most commonly used (53.6%). Patients with immunotype C tumors more frequently underwent the classic technique, whereas those with immunotypes A and B tumors were more often treated with the modified approach, with the difference being statistically significant (*p* = 0.005). Both pancreatic fistulas and acute pancreatitis were complications most commonly developed by the patients who present a cold tumor (*p* < 0.001). Biliary fistulas were extremely rare (3.6% of the total number of cases). Regarding tumor staging, no statistically significant differences were observed, with the vast majority of patients classified as stage IIB, regardless of tumor type. In terms of the TNM classification, most patients were categorized as T3, N1 and Mx (analyzed independently).

### 3.3. Analysis of the Complications

The data from Table 3 revealed significant differences in the percentage of patients developing pancreatic fistulas and acute pancreatitis, when stratified by their immunotypes. Specifically, patients with immunotype C pancreatic ductal adenocarcinoma exhibit considerably higher rates of both pancreatic fistulas and acute pancreatitis.

Risk factors were analyzed using a stepwise logistic regression model (both forward and backward directions), with AIC as the selection criterion (Table 4). The initial model for both pancreatic fistulas and acute pancreatitis included the respective pathology as the outcome, with immunotype, stage, grade, age, and sex as covariates.

For pancreatic fistulas, the selected model (with the lowest AIC) identified tumor immunotype, grade, and sex as significant predictors. This model explains approximately 58% of the variation in pancreatic fistula development. However, no statistically significant associations were found for stage. Male sex was associated with a 1.8-fold increased risk of developing pancreatic fistulas (*p* = 0.037), with 75% of fistulas complicating pancreatic adenocarcinoma arising from this group. Immunotypes B and C significantly increased the risk of this complication by factors of 3.68 (*p* = 0.002) and 3.94 (*p* = 0.001), respectively.

To further assess these risks, a Monte Carlo simulation with 10,000 samples was conducted. The simulations yielded the following estimated probabilities of fistula formation for each immunotype: 2.5% for immunotype A, 25% for immunotype B, and 28% for immunotype C.

Regarding acute pancreatitis, the results are unclear. The stepwise regression modeling stopped at the initial step (with the lowest AIC); therefore, all the variables (type, stage, grade, age, and sex) were considered valuable for predicting the risk of developing acute pancreatitis. Yet, only age had a significant *p*-value (0.04), while type C histological type had a marginally significant value of *p* (0.09). With all of this lack of significance, the model seems to be predicting a chosen outcome in proportion of about 88%. This could be due to the data being insufficient to properly analyze the risk factors of developing acute pancreatitis in patients with pancreatic ductal adenocarcinoma. Further analysis of this matter has not been conducted.

### 3.4. Survival Analysis

**Figure 6:**
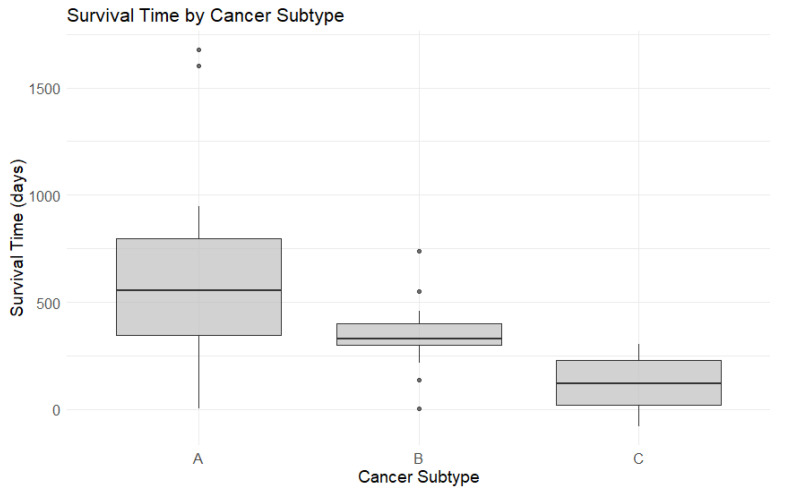
Box plots for survival time by cancer subtype.

**Figure 7:**
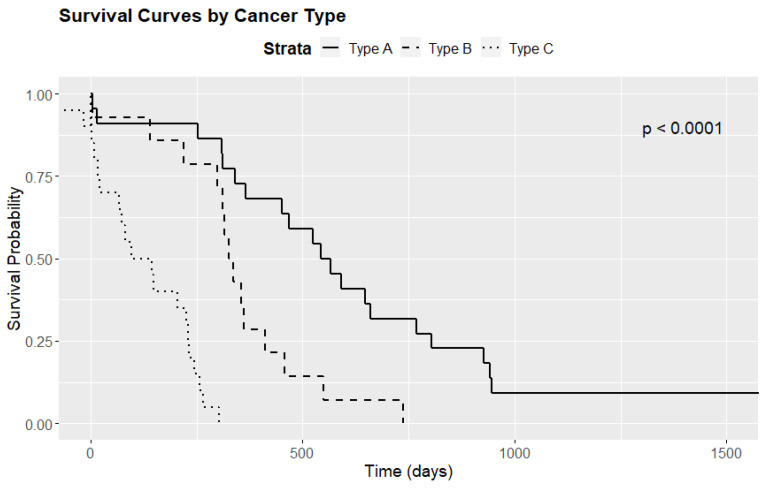
Survival curves by cancer subtype.

There was a statistically significant difference in median survival times according to tumor immunotype (*p* < 0.001). Specifically, patients with immunotype C tumors had a median survival time of only 120.5 days, compared to 553.5 days for those with immunotype A and 331.5 for immunotype B tumors. Figure 6 presents boxplots of the survival times, while Figure 7 displays Kaplan–Meier survival curves. Table 5 highlights the overall survival rates at 28 days, 1 year, 2 years, and 3 years, computed based on the survival regression model.

Further analysis involved pairwise comparisons using the log-rank test. The results were as follows: A vs. B (*p* = 0.008), B vs. C (*p* < 0.001), and A vs. C (*p* < 0.001). These findings indicate statistically significant differences in survival times among the three immunotypes, suggesting that the variation in survival is both substantial and potentially clinically meaningful.

## 4. Discussion

Pancreatic ductal adenocarcinoma is an aggressive neoplasm with high mortality, and systemic therapy is an essential component of multimodal therapy [20]. Studies show a primary and secondary resistance to current therapy and therefore there is a need to develop new therapeutic modalities with oncologic benefit [21]. Although immunotherapy has revolutionized the treatment of different types of neoplasms, studies have shown that pancreatic ductal adenocarcinoma is unresponsive or poorly responsive to these therapies [22]. Therefore, we believe focusing on the tumor microenvironment is of interest in order to identify its role in the resistance to different systemic therapies and as a target for new effective therapeutic modalities.

Pancreatic adenocarcinoma lacks excessive mutations required by the host immune system to recognize tumor cells by tumor-specific antigens and is considered an immunologically “cold” tumor. This low immunogenicity results in an insufficient presentation of the cancer antigen, leading to an absent or very weak immune response as well as an inadequate T-cell influx. The tumor also programs the surrounding tissue and activates immune cells to produce a dense, fibroblastic peritumoral environment with limited antitumor immunity [23,24,25]. In the selected group, an approximately equal incidence for the hot and cold immunotype (approximately 30%) was observed. The differences compared to the medical literature could be explained by the presence of the small number of patients included in this study.

Carcinogenesis, but also the invasion and dissemination potential of pancreatic ductal adenocarcinoma, is a complex process that must be understood through the interactions between tumor cells and tumor microenvironment cells. The pancreatic cancer microenvironment consists of malignant cells, endothelial cells, immune cells, and pancreatic stellate cells [19,26]. The tumor microenvironment also includes the extracellular matrix, local microbiota as well as the biochemical, biophysical, and bioelectric conditions of the stroma and interstitial fluid that exist around the tumor [27,28,29]. Investigating the tumor microenvironment can lead to a better understanding of the mechanisms involved in pancreatic carcinogenesis and allows opportunities for new treatment challenges.

The tumor immune microenvironment has a prognostic significance in pancreatic ductal adenocarcinoma [30,31]. The present study suggests that the immune subtype of pancreatic adenocarcinoma plays an important role in overall survival, as well as in the risk of complications.

The immune cells represent approximately 50% of the total number of cells in ductal adenocarcinoma, but only a small subgroup are tumor suppressive cells [32]. The cells of the immune microenvironment that play important roles in the process of carcinogenesis and tumor progression are lymphocytes, macrophages, neutrophils, antigen-presenting dendritic cells, and natural killer T lymphocytes [30]. Numerous studies show that tumor infiltrating lymphocytes have a favorable prognosis and increased survival of the patients [31,32,33,34,35]. In the selected group, a predominance of lymphocytes in the tumor microenvironment was identified, with a rate of 60%, 55%, and 90%, respectively, for the three immunotypes. Macrophages, plasma cells, neutrophil granulocytes, eosinophils, and antigen-presenting dendritic cells were also identified.

Carstens et al. [36] performed an immunohistochemical study and found that the tumor immune microenvironment of pancreatic ductal adenocarcinoma is predominantly constituted by CD8 positive cytotoxic T lymphocytes and CD4 positive helper T lymphocytes, proving that they are an independent indicator of positive prognosis. They correlated the proximity of CD8 positive T lymphocytes to tumor cells with better survival. This result is also confirmed by other studies that show that patients with numerous positive CD8 lymphocytes in the tumor microenvironment have a better prognosis, with increased long-term survival [37,38,39]. In contrast, in the present study, a predominance of CD20-positive B lymphocytes in the tumor immune microenvironment was observed, with a large increase (twofold) from subtype A, with higher survival, to subtype C, with lower survival.

CD3-positive lymphocytes also correlated with good survival in a multivariate analysis study by Miksch et al. [33]. The data from the literature overlap with those obtained in the selected group. In subtype A of pancreatic ductal adenocarcinoma, a rate of 30% for CD3 positive T lymphocytes was observed, compared to subtype B (15%) and C (5%), respectively.

Macrophages are cells derived from circulating monocytes, being recruited at the site of the tumor by chemokines and growth factors, forming a heterogeneous cell population characterized by plasticity and the ability to switch between anti-tumor M1 and pro-tumor M2 phenotypes, depending on conditions in the tumor microenvironment and activation signals [40,41]. Some studies show that it represents one of the most abundant immune cell subtypes in pancreatic cancer [42]. Griesmann et al. conducted a study on mice showing that the decrease in macrophages leads to a decrease in the risk of lung and liver metastases [43]. In the present study, a statistically significant correlation between the presence of macrophages and the appearance of liver and lung metastases was not observed, but an increased rate of macrophages in subtypes A and B was identified associated with increased overall survival. In contrast, subtype C, which was associated with poor survival, did not show macrophages in the tumor immune microenvironment.

Neutrophils represent an essential component of the innate immune system and have increased anticancer activity, being able to induce phagocytosis and direct cytotoxic elimination of tumor cells [40]. In the tumor immune microenvironment, neutrophils can be distinguished by their cytokine status, activation, and effects on tumor cells. Thus, the N1 subtype, regulated by interferon α, exerts antitumor effects through cytotoxic action against tumor cells, as well as immunosuppressive effects at the level of the tumor immune microenvironment, both through the recruitment and activation of T helper lymphocytes. The N2 subtype is induced by TGF-β and shows a switch to a pro-tumor phenotype that promotes tumor progression by remodeling the tumor microenvironment [30,41]. Neutrophils contribute to tumor invasion and distant dissemination by secreting VEGF and metalloproteinase-9 and stimulating tumor angiogenesis. Thus, the high number of neutrophils could provide a survival advantage for neoplastic cells, leading to recurrences for patients with pancreatic adenocarcinoma. Prevention of neutrophil maturation and migration by the tyrosine kinase inhibitor Lorlatinib has been shown to abrogate pancreatic carcinogenesis and metastasis [44,45]. The results of the present study were partially superimposed on the data from the literature. Thus, in the selected group a low rate of neutrophil granulocytes was observed in subtypes A and B, with better survival, while in subtype C the rate was 0%.

Antigen-presenting dendritic cells regulate anti-tumor immune responses by activating CD8 and CD4 positive T lymphocytes through the Major Histocompatibility Complex. They infiltrate pancreatic tumors, and their abundance is associated with inhibition of disease progression [44,46]. In the selected group, a 5% rate of dendritic cells was noted only in the hot subtype of pancreatic ductal adenocarcinoma.

Mast cells have been present in the solid tumor microenvironment of numerous malignancies and have been found to be a favorable prognostic factor in some malignant tumors, such as ovarian and esophageal carcinoma, and also in lymphoma. However, in other neoplasms, such as lung, breast, and gastric carcinoma, as well as in melanoma, tumor-associated mast cells were associated with a poor prognosis [47]. In the pancreatic adenocarcinoma, mast cells are involved in the immune response regulation and tumor angiogenesis via releasing numerous bioactive substances such as extracellular matrix degradation factors and pro-angiogenic factors [48]. Mast cell migration in the tumor microenvironment is induced by the expression of several growth factors, proinflammatory cytokines, and molecules, such as chemokines, prostaglandins SCF, TGF, TNF, and FES kinase [49]. Guo et al. found in an immunohistochemical study that mast cell levels are increased in pancreatic cancer tissues compared to those in matched peritumorally tissues [48], and the results from the current study showed a favorable association of mast cell levels with the survival rate.

Downregulation of CD5 positive lymphocytes on the tumor immune microenvironment enhances antitumor T cell activity [50]. No immunohistochemical studies of CD5-positive lymphocytes in pancreatic adenocarcinomas could be identified in the medical literature. Our study showed a high rate of CD5-positive T lymphocytes in tumors with median survival time of 553.5 days, as well as a decrease in tumors with median survival time of only 120.5 days.

Pancreatic fistula is one of the most serious complications after surgery, with the rate varying widely from 2% to well over 20% [51,52]. For pancreatic fistulas, the selected model identified tumor immunotype, grade, and sex as significant predictors. This model explains approximately 58% of the variation in pancreatic fistula development. However, no statistically significant associations were found for stage. Male sex was associated with a 1.8-fold increased risk of developing pancreatic fistulas, with 75% of fistulas complicating pancreatic adenocarcinoma arising from this group. Immunotypes B and C significantly increased the risk of this complication by factors of 3.68 and 3.94, respectively. Regarding acute pancreatitis, the results are unclear. The stepwise regression modeling stopped at the initial step; therefore, all the variables (immunotype, stage, grade, age, and sex) were considered valuable for predicting the risk of developing acute pancreatitis. Yet, only age had a significant *p*-value (0.04), while type C histological type had a marginally significant value of *p* (0.09). With all of this lack of significance, the model seemed to predict the chosen outcome in proportion of about 88%. This could be due to insufficient data to properly analyze the risk factors of developing acute pancreatitis in patients with pancreatic ductal adenocarcinoma. Further analysis of this matter has not been conducted.

Pancreatic ductal adenocarcinoma is characterized by a fibrotic stroma with the dense extracellular matrix. The desmoplastic stroma is a hallmark of pancreatic ductal adenocarcinoma, and represents a physical barrier with immunosuppressive roles, which limits the infiltration of immune cells and directly suppresses their cytotoxic function. This fibrous stroma also prevents the penetration of chemotherapy agents into the tumor microenvironment [53,54]. Our results were superimposable over the literature data, fibrous stroma being a favorable prognostic factor. A histochemical study of desmoplasia suggested that immunotype A tumors with better survival rates presented low areas of fibrosis, while marked fibrosis in immunotype C tumors was associated with a marked decrease in survival rates.

## 5. Conclusions

The tumor immune microenvironment has a prognostic significance in pancreatic ductal adenocarcinoma. The identification of the immunotype of pancreatic ductal adenocarcinoma can be a predictive factor for the occurrence of complications such as pancreatic fistula as well as for overall survival.

## Figures and Tables

**Table 1:** Data related to the antibodies used for immunohistochemical reactions.

Antibody	Substrate	Clone	Dilution	Cell Type
LCA ^1^	Monoclonal, mouse	X16/99	1:40	Lymphocyte
CD3 ^2^	Monoclonal, mouse	LN10	1:500	T lymphocyte
CD4 ^3^	Monoclonal, mouse	4B12	1:100	T helper lymphocyte
CD8 ^4^	Monoclonal, mouse	4B11	1:500	T cytotoxic lymphocyte
CD20 ^5^	Monoclonal, mouse	L26	RTU ^6^	B lymphocyte
CD68 ^7^	Monoclonal, mouse	514H12	1:100	Macrophage
CD1a ^8^	Monoclonal, mouse	MTB1	RTU	Dendritic cell
CD117 ^9^	Monoclonal, rabbit	EP10	1:200	Mast cell

^1^ LCA (leucocyte common antigen); ^2^ CD3 (cluster of differentiation 3); ^3^ CD4 (cluster of differentiation 4); ^4^ CD8 (cluster of differentiation 8); ^5^ CD20 (cluster of differentiation 20); ^6^ RTU (Ready-to-use); ^7^ CD68 (cluster of differentiation 68); ^8^ CD1a (cluster of differentiation 1a); ^9^ CD117 (cluster of differentiation 117).

**Table 2:** Detailed immunohistochemical profile of the tumor immune microenvironment.

Tumor Type	ImC ^1^	Ly ^2^ LCA+	LyB CD20+	LyT CD4+	T CD8+	T CD3+	T CD5+	Mph ^3^ CD68+	Mast ^4^ CD117+	DCs ^5^ CD1a+	Other Cells	MTS ^12^
Type A	+++ ^6^	60%	35%	10%	5%	30%	20%	20%	10%	5%	5% GN ^7^	+
Type B	++ ^8^	55%	15%	10%	40%	15%	20%	20%	5%	0%	10% Pl ^9^, 5% GN, 5% Eo ^10^	++
Type C	+ ^11^	90%	60%	5%	20%	5%	10%	<0.1%	<0.1%	0%	10% Pl	+++

ImC ^1^ immune cells; Ly ^2^ lymphocytes; Mph ^3^ macrophages; Mast ^4^ mast cells; DCs ^5^ dendritic cells; +++ ^6^ marked; GN ^7^ neutrophil granulocytes; ++ ^8^ moderate; Pl ^9^ plasma cells; Eo ^10^ eosinophils; + ^11^ discret; MTS ^12^ Masson’s trichrome staining.

**Table 3:** Descriptive statistics.

Variable		All Patients (*n* = 56)	Type A (*n* = 22)	Type B (*n* = 14)	Type C (*n* = 20)	*p*-Value *^,^**
Age (years) ^(a)^		64 (59–70)	64 (61.25–67)	59 (53.5–69.75)	65 (61–71.25)	0.633
Survival time (days) ^(a)^		310.5 (142.8–529.2)	553.5 (346.2–795.8)	331.5 (301.5–399.2)	120.5 (19.25–229.75)	<0.001 **
Sex (female) ^(b)^		24 (42.9%)	11 (50%)	3 (21.4%)	10 (50%)	0.174
Preoperatory biliary stent ^(b)^		29 (51.8%)	11 (50%)	9 (64.3%)	9 (45%)	0.529
Classic technique ^(b)^		26 (46.4%)	6 (27.27%)	5 (37.71%)	15 (75%)	0.005 **
Modified technique ^(b)^		30 (53.6%)	16 (72.7%)	9 (64.3%)	5 (25%)	
Pancreatic fistula ^(b)^		20 (35.7%)	1 (4.55%)	8 (57.14%)	11 (55%)	<0.001 **
Biliary fistula ^(b)^		2 (3.6%)	2 (9.09%)	-	-	0.2
Acute pancreatitis ^(b)^		20 (35.7%)	1 (4.55%)	3 (21.43%)	16 (80%)	<0.001 **
Stage ^(b)^						
	IA	4 (7.1%)	2 (9.1%)	-	2 (10%)	0.08
	IB	4 (7.1%)	3 (13.6%)	1 (7.1%)	-	
	IIA	11 (19.6%)	1 (4.6%)	3 (21.4%)	7 (35%)	
	IIB	29 (51.8%)	13 (59.1%)	10 (71.4%)	6 (30%)	
	III	4 (7.1%)	2 (9.1%)	-	2 (10%)	
	IV	4 (7.1%)	1 (4.6%)	-	3 (15%)	
T1 ^(b)^		8 (14.3%)	4 (18.2%)	-	4 (20%)	0.09
T2 ^(b)^		14 (25%)	7 (31.8%)	3 (21.4%)	4 (20%)	
T3 ^(b)^		31 (55.4%)	8 (36.4%)	11 (78.6%)	12 (60%)	
T4 ^(b)^		3 (5.4%)	3 (13.64%)	-	-	
N0 ^(b)^		14 (25%)	2 (9.1%)	3 (21.4%)	9 (45%)	0.144
N1 ^(b)^		31 (55.4%)	13 (59.1%)	9 (64.3%)	9 (45%)	
N2 ^(b)^		1 (1.8%)	1 (4.6%)	-	-	
NX ^(b)^		10 (17.9%)	6 (27.3%)	2 (14.3%)	2 (10%)	
M0 ^(b)^		17 (30.4%)	6 (27.3%)	3 (21.4%)	8 (40%)	0.226
M1 ^(b)^		4 (7.1%)	1 (4.6%)	-	3 (15%)	
MX ^(b)^		35 (62.5%)	15 (68.2%)	11 (78.6%)	9 (45%)	

^(a)^ median (IQR), Kruskall–Wallis test; ^(b)^ counts (percentages), either asymptotic Chi-Square statistical test; ^(^*^)^—statistically significant, *p* < 0.05; ^(^**^)^—statistically significant, *p* < 0.01.

**Table 4:** Summary of the logistic regression models with the best statistical fit for predicting pancreatic fistulas and acute pancreatitis.

Variable	OR	SD	95% CI	*p* Value
Pancreatic fistulas ~ type + grade + age + sex ^(a)^ Nagelkerke R^2^ = 0.58				
Intercept	−4.01	1.66	−7.9, −1.02	0.015 *
Immunotype B	3.68	1.23	1.59, 6.81	0.003 **
Immunotype C	3.94	1.23	1.89, 7.07	0.001 **
Grade G2	−0.3	1.43	-	0.834
Grade G3	−18.84	1906	-	0.992
Sex (Male)	1.81	0.86	1.22, 38.94	0.037 *
Acute pancreatitis ~ type + stage + grade + age + sex ^(b)^ Nagelkerke R^2^ = 0.88				
Intercept	−88.82	10178	-	0.993
Immunotype B	7.25	4.28	1.56, 20.81	0.09
Immunotype C	44.98	6487	-	0.995
Stage IB	46.04	6487	-	0.994
Stage IIA	44.21	6487	-	0.995
Stage IIB	39.45	6487	-	0.995
Stage III	1.27	6.46	-	0.844
Stage IV	0.39	2.99	-	0.895
Grade G2	24.74	4529	-	0.995
Grade G3	25.3	4259	-	0.996
Age	0.29	0.142	0.072, 0.68	0.042 *
Sex (Male)	−4.84	3.21	-	0.132

^(^*^)^—statistically significant, *p* < 0.05; ^(^**^)^—statistically significant, *p* < 0.01. ^(a)^—initial model: pancreatic fistulas ~ immunotype + stage + grade + age + sex; ^(b)^—initial model: acute pancreatitis ~ immunotype + stage + grade + age + sex. Abbreviations: CI—confidence intervals; OR—odds ratio; SD—standard deviation.

**Table 5:** Overall survival rates at 28 days, 1, 2, and 3 years.

Variable	All Patients (*N* = 56)	Type A (*N* = 22)	Type B (*N* = 14)	Type C (*N* = 20)
OS 28 days	84%	91%	93%	75%
OS 365 days	34%	68%	29%	-
OS 730 days	13%	32%	7%	-
OS 1095 days	3.6%	9%	-	-

Abbreviations: OS—overall survival.

## Data Availability

The data that support the funding on this study are available from the corresponding author upon reasonable request.

## Supplementary Materials

The following supporting information can be downloaded at: https://www.mdpi.com/article/10.3390/diagnostics15050646/s1, Figure S1: Microscopical aspects in immunotype A, B and C tumors, HE staining, imunohistochemical and histochemical staining, ob.10×; MTS: Masson’s trichrome staining.
