# Supplementary material for: Immunohistochemical Evaluation of the Tumor Immune Microenvironment in Pancreatic Ductal Adenocarcinoma
Source: Diagnostics (Basel). 2025 Mar 6;15(5):646. doi: 10.3390/diagnostics15050646 (PMC11899021; doi:10.3390/diagnostics15050646)
Supplement: Supplementary file 1 [file diagnostics-15-00646-s001.zip › diagnostics-3492092-supplementary.pdf]

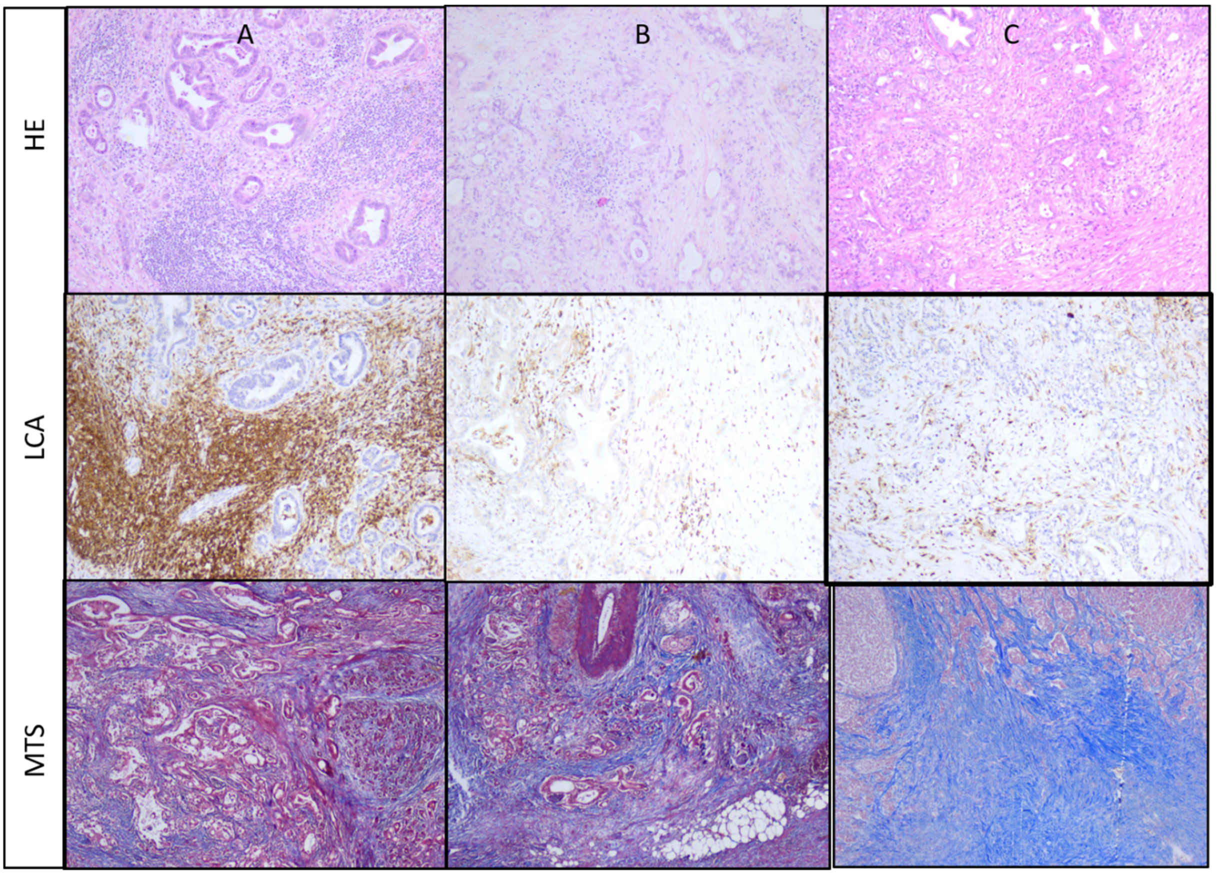

Supplemental Figure S1: Microscopical aspects in immunotype A, B and C tumors, HE staining, imunohistochemical and histochemical staining, ob.10x; MTS: Masson’s trichrome staining.
